# Electroacupuncture improves upper-limb motor function and modulates sensorimotor network connectivity in subacute stroke: a randomized controlled trial

**DOI:** 10.3389/fneur.2026.1780918

**Published:** 2026-06-19

**Authors:** Mingfen Li, Weigeng Zou, Genggeng Xie, Man Li, Su Zheng, Haifeng Li

**Affiliations:** Department of Neurorehabilitation, Hubei Provincial Clinical Research Center for Central Nervous System Repair and Functional Reconstruction, Taihe Hospital, Hubei University of Medicine, Shiyan, Hubei, China

**Keywords:** electroacupuncture, electroencephalography, neuroplasticity, stroke rehabilitation, upper-limb

## Abstract

**Objective:**

Electroacupuncture (EA) has been shown to facilitate post-stroke motor recovery; however, the underlying neurophysiological mechanisms remain insufficiently understood. This study aimed to investigate how EA modulates sensorimotor network (SMN) connectivity and to explore its relationship with upper-limb motor recovery in patients with subacute stroke.

**Methods:**

Fifty patients with subacute stroke were randomly assigned to the EA group or sham EA (SEA) group. All participants received 18 treatment sessions over 3 weeks in addition to standard rehabilitation. Upper-limb motor function and daily living ability were assessed before and after treatment. Resting-state electroencephalography (EEG) was recorded before and after the intervention. Functional connectivity (FC) within the SMN was calculated using phase-locking value (PLV) in the alpha (8–13 Hz) and beta (13–30 Hz) bands. Graph-theoretical metrics were analyzed, and partial least squares regression (PLSR) was used to evaluate associations between FC alterations and motor improvement.

**Results:**

After treatment, the EA group showed significantly greater improvement in upper-limb motor function than the SEA group, as indicated by a significant group × time interaction for the Fugl-Meyer Assessment for Upper Extremity (FMA-UE) and greater between-group gains in change scores (*p* < 0.05). For the Modified Barthel Index (MBI), significant main effects of time and group were observed, but the group × time interaction was not significant, although Week 3 scores were higher in the EA group (*p* < 0.05). EEG analysis showed that alpha-band SMN FC significantly increased after EA, whereas no significant changes were observed in the SEA group (*p* < 0.05). Between-group comparisons revealed higher post-treatment alpha-band FC in the EA group, predominantly involving interhemispheric connections (*p* < 0.05). In the EA group, PLSR explained 68.7% of the variance in motor improvement (*R^2^* = 0.687; RMSE = 7.68), whereas no valid predictors were identified in the SEA group. No significant differences were found in global network topology metrics (*p* > 0.05).

**Conclusion:**

EA improved upper-limb motor recovery in patients with subacute stroke and was accompanied by selective enhancement of alpha-band functional connectivity within the sensorimotor network. These findings suggest that EA may promote post-stroke motor recovery through frequency-specific reorganization of recovery-relevant sensorimotor circuits.

**Clinical trial registration:**

https://www.chictr.org.cn/bin/project/edit?pid=199590, Chinese Clinical Trial Registry (Registration number: ChiCTR2300077420).

## 1 Introduction

Stroke remains one of the leading causes of death and disability worldwide (1). Upper-limb motor dysfunction is among the most prevalent and disabling sequelae. Approximately 50–80% of patients exhibit upper-limb impairment in the acute phase and 40–50% continue to show deficits in fine motor control during the subacute stage (2). These impairments restrict independence and social participation, underscoring the need for reproducible interventions and objective neurophysiological markers that reflect recovery processes.

Electroacupuncture (EA), which integrates acupuncture with controlled electrical stimulation, is increasingly utilized as an adjunctive intervention for post-stroke motor rehabilitation (3). Compared with manual acupuncture, EA may provide more stable and controllable stimulation across repeated treatment sessions (4). However, EA effects are likely influenced by the specific configuration of stimulation parameters, rather than by electrical stimulation alone. Previous stroke rehabilitation trials have applied diverse EA configurations, utilizing various frequencies (e.g., continuous 2 Hz or dense-disperse 2/15 Hz) and current intensities (e.g., 1–3 mA titrated to patient tolerance) across multi-session courses (5). Such parameter heterogeneity limits cross-study comparability, weakens reproducibility, and hinders the development of precision protocols. Accordingly, identifying objective biomarkers that capture parameter-dependent neural responses is an important need to optimize EA prescriptions and improve therapeutic efficacy in stroke motor rehabilitation.

Previous brain imaging studies using modalities such as functional magnetic resonance imaging (fMRI), functional near-infrared spectroscopy (fNIRS), and electroencephalography (EEG) have suggested that EA can modulate cortical activity and neuroplasticity in patients with stroke. Hemodynamic imaging studies have mainly highlighted EA-related changes in regional cortical activation and interregional network recruitment. Specifically, fMRI investigations have shown that acupuncture is associated with widespread changes in motor-related brain activity, involving the cerebellum, basal ganglia, frontal regions, and precuneus in patients with post-stroke motor dysfunction (6). In parallel, fNIRS studies have further shown that acupuncture can enhance ipsilesional primary motor cortex activation and modulate directed functional connectivity between premotor, sensory, and motor-related regions, suggesting a potential role in remodeling of the sensorimotor network (SMN) after stroke (7). However, findings across modalities remain heterogeneous, and many imaging approaches have limited ability to capture rapid, frequency-specific neural coupling underlying post-stroke network reorganization. These limitations support the need for a network-level EEG approach to characterize EA-related cortical modulation more directly.

EEG provides a portable, high-temporal-resolution window for examining dynamic neural communication after stroke (8). By recording real-time cortical oscillations, EEG enables the quantification of functional connectivity, such as phase synchronization, across spatially distributed cortical nodes (9). This sensitivity to frequency-specific neural coupling makes EEG particularly suitable for evaluating neuromodulatory interventions such as EA, especially in the subacute phase, which offers heightened neuroplastic responsiveness and greater clinical stability. Preliminary EEG studies indicate that EA modulates cortical oscillations, alters functional coupling, and influences network topology in patients with stroke (10–12). Critically, these oscillatory modulations are most prominent within the alpha (8–13 Hz) and beta (13–30 Hz) bands that are closely related to post-stroke motor recovery (13) and EA-related EEG modulation (14). In addition, resting-state analysis can characterize intervention-related network reorganization while minimizing task-performance confounds, which is particularly important in patients with heterogeneous motor impairment severity.

Within the large-scale networks involved in motor recovery, the SMN is of particular interest because it supports motor planning, motor execution, and somatosensory feedback integration through coordinated activity across motor- and somatosensory-related cortical regions (15). Stroke typically disrupts SMN organization, manifesting as reduced ipsilesional connectivity, interhemispheric imbalance, and aberrant neural synchronization, all of which are associated with motor impairment and recovery potential (16). Given that EEG is well suited to characterizing frequency-specific network coupling, SMN connectivity represents a particularly relevant target for investigating EA-related cortical modulation in post-stroke rehabilitation. In our previous work (17), EEG-derived SMN connectivity changes following EA were associated with upper-limb motor recovery, suggesting that frequency-specific SMN features may serve as candidate biomarkers of treatment responsiveness. However, the current EA-EEG literature remains limited by relatively small sample sizes, heterogeneous stimulation protocols and stroke stages, and a lack of sham-controlled randomized studies. Whether EA induces frequency-specific SMN reorganization that is directly associated with behavioral improvement therefore remains unclear.

Against this background, we conducted a sham-controlled randomized study in patients with subacute stroke, integrating behavioral assessment with resting-state EEG network analysis to investigate the electrophysiological mechanisms of EA. Specifically, we aimed to evaluate the efficacy of EA as an adjunct to conventional rehabilitation and determine its modulatory effects on SMN functional connectivity in the alpha and beta bands. We hypothesized that, compared with sham stimulation, active EA would produce greater upper-limb motor improvement accompanied by frequency-specific SMN reorganization, and that these network-level alterations could predict inter-individual recovery outcomes using multivariate modeling. Ultimately, this study provides objective neurophysiological evidence to elucidate the network-level mechanisms of EA, supporting its precise application in post-stroke motor rehabilitation.

## 2 Methods

### 2.1 Study design

This randomized controlled trial was conducted in accordance with the Consolidated Standards of Reporting Trials (CONSORT) and Standards for Reporting Interventions in Controlled Trials of Acupuncture (STRICTA) guidelines (18). Consecutive inpatients clinically diagnosed with ischemic stroke were recruited from the Department of Neurological Rehabilitation, Taihe Hospital (Shiyan, China), between November 10, 2023, and February 29, 2024. Recruitment was achieved through on-site presentations and poster advertisements at the hospital. The study protocol was approved by the Ethics Committee of Shiyan Taihe Hospital and registered in the Chinese Clinical Trial Registry (ChiCTR2300077420). All procedures adhered to the principles of the Declaration of Helsinki, and written informed consent was obtained from all participants or their legal representatives prior to enrollment.

### 2.2 Participant recruitment

The inclusion criteria were as follows: (1) age between 30 and 70 years; (2) first-ever stroke, diagnosed according to the criteria established at the Fourth National Conference on Cerebrovascular Diseases (1995); (3) stroke lesions confined primarily to subcortical regions; (4) severe upper-limb motor impairment, defined as Brunnstrom stage I or II of the affected upper-limb or hand; (5) stroke onset between 15 days and 3 months before enrollment; (6) adequate cognitive function to follow simple instructions, without major auditory comprehension deficits; (7) right-handedness and ability to maintain a seated posture for at least 30 min; (8) no previous acupuncture treatment; and (9) provision of informed consent.

The exclusion criteria were as follows: (1) contraindications to electroacupuncture; (2) life-threatening comorbidities with a greater impact on quality of life than post-stroke limb dysfunction; (3) epilepsy or psychiatric disorders requiring medication; and (4) unwillingness to comply with treatment or assessments.

### 2.3 Sample size

Sample size estimation was performed using G*Power software (version 3.1.9.6). Based on preliminary data from 22 stroke patients in a pilot trial, the effect size for changes in Fugl-Meyer Assessment for Upper Extremity (FMA-UE) scores was calculated to be 0.94. Assuming a two-tailed *α* of 0.05 and *a* power of 0.80, and equal group allocation (1:1), a minimum of 20 participants per group was required using the Wilcoxon-Mann–Whitney test. Considering a 20% dropout rate, the final sample size was 50 participants.

### 2.4 Randomization and blinding

Participants were randomly assigned in a 1:1 ratio to either the EA or sham EA (SEA) group. The randomization sequence was generated by an independent researcher using SPSS version 26.0 and concealed in sequentially numbered, opaque, and sealed envelopes. Owing to the procedural characteristics of acupuncture, treatment allocation was masked for all personnel except the acupuncturists. The participants, outcome assessors, and data analysts remained blinded throughout the study to ensure methodological rigor.

### 2.5 Interventions

Both groups received standard motor rehabilitation combined with distinct acupuncture protocols applied to the affected upper limb. Specifically, the standard motor rehabilitation program consisted of comprehensive physical therapy and occupational therapy tailored to each patient’s clinical condition. These conventional rehabilitation sessions were administered for approximately 6 h per day, 6 days per week, in both groups. All acupuncture treatments were performed by two licensed acupuncturists with over 5 years of clinical experience who were trained in standardized procedures. Training included acupoint selection, localization, stimulation parameters, and *deqi* evaluation using the Chinese version of the Massachusetts General Hospital Acupuncture Sensation Scale (C-MASS) (19). Treatments were administered once daily, 6 days per week, for three consecutive weeks. To maintain blinding, participants were treated individually in separate rooms, and the affected upper limb was covered during stimulation.

#### 2.5.1 EA intervention

**Figure 1:**
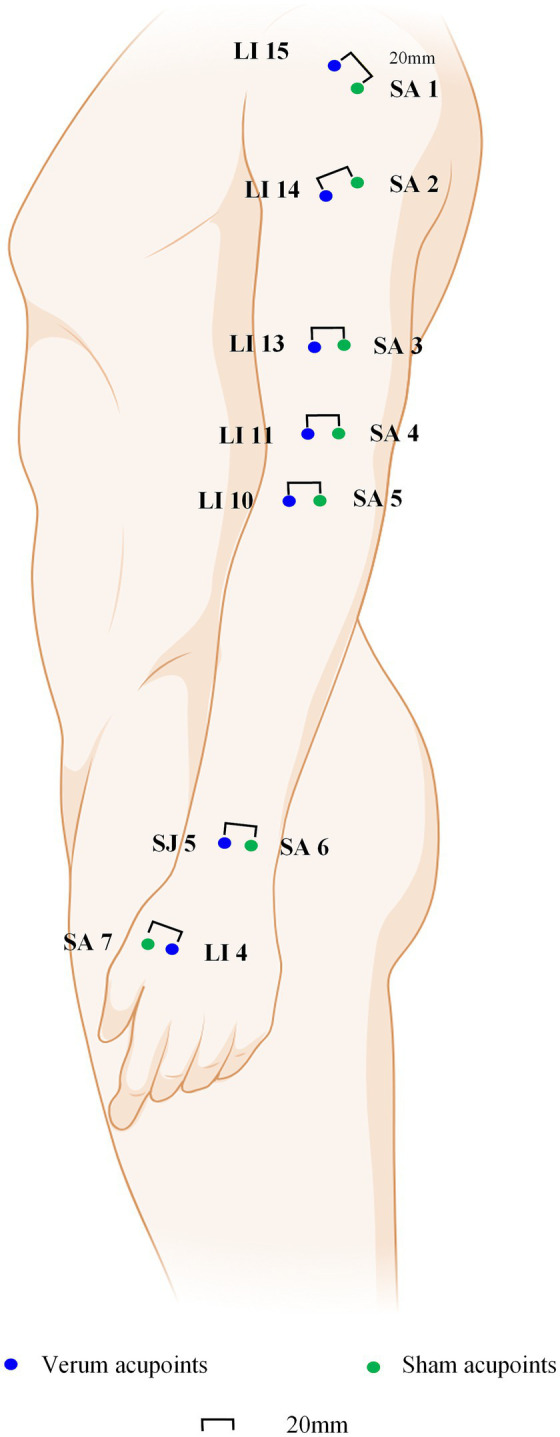
Acupoint locations in the EA and SEA groups. Blue circles indicate verum acupoints used in the EA group, and green circles indicate corresponding sham acupoints used in the SEA group. EA, electroacupuncture; SEA, sham electroacupuncture. Created in BioRender. Li, M (2026) https://BioRender.com/o94qti5. License/Agreement number: FB29TG4X46.

Acupoint selection followed the classical principle of “treating flaccidity by stimulating the Yangming meridian” (20). Seven acupoints were selected on the affected upper limb (Figure 1): Jianyu (LI15), Binao (LI14), Shouwuli (LI13), Quchi (LI11), Shousanli (LI10), Waiguan (SJ5), and Hegu (LI4). Localization followed the national standard *Nomenclature and location of meridian points*: GB/T 12346–2021 (21).

After skin disinfection with povidone-iodine, sterile disposable needles (0.30 mm× 40 mm, Hwato, Suzhou Medical Appliance Factory, China) were inserted perpendicularly to a depth of 10–15 mm. *Deqi* was confirmed using the C-MASS scale. Needles at LI11, LI13, LI10, and LI4 were connected to an EA device (Model 6,805-D, Shantou Medical Equipment Co., China). Regarding electrode polarity, the positive leads were connected to the proximal acupoints (e.g., LI10 or LI13), while the negative leads were connected to the distal acupoints (e.g., LI4 or LI11) to ensure standardized current direction. The device delivered a discontinuous wave at 2 Hz. The current intensity was individually titrated within a specific range of 1–3 mA. It was gradually increased until slight needle vibration occurred without visible muscle contraction or patient discomfort. Each session lasted 30 min.

#### 2.5.2 Sham EA intervention

Following previous research protocols (22), non-acupoint sites approximately 20 mm lateral to the verum EA acupoints were used for the SEA group. These sites were unrelated to any recognized meridian or acupoint. After disinfection, shorter disposable needles (0.30 mm× 13 mm) were inserted perpendicularly to a depth of 3–5 mm, sufficient to penetrate the skin without manual manipulation. At this shallow depth, the needles only penetrated the epidermal and superficial dermal layers, deliberately avoiding the subcutaneous connective tissue and deeper muscular layers. Different needle lengths and insertion depths were intentionally used to maintain participant blinding while minimizing deep-tissue mechanosensory afferent activation in the sham condition.

*Deqi* was assessed using the C-MASS scale, and a score of zero or reports of sharp pain were interpreted as the absence of *deqi*. Subsequently, the needles were connected to an internally short-circuited EA device identical in appearance to that used in the EA group (2 Hz, discontinuous wave). Although the indicator light remained illuminated, no current was delivered. To maintain blinding, all participants were informed that the stimulation intensity might be below the sensory threshold.

### 2.6 Outcome measures

Baseline behavioral assessments and electroencephalography (EEG) recordings were conducted within 3 days after enrollment. Clinical assessments were subsequently repeated at 1-week intervals. The final clinical evaluations, alongside the second EEG recording, were strictly completed within 72 h following the final treatment session at Week 3. All evaluations were performed by the same trained physician, who was blinded to group allocation and underwent standardized instruction in the administration of all scales.

#### 2.6.1 Primary outcomes

Upper-limb motor function was assessed using the FMA-UE at baseline, Week 1, Week 2, and Week 3. The FMA-UE provides a quantitative evaluation of motor performance. Specifically, it is a highly validated, stroke-specific performance-based index comprising 33 items that assess reflexes, flexor and extensor synergies, wrist and hand function, and coordination (23). Each item is scored on a 3-point ordinal scale (0 = cannot perform, 1 = performs partially, 2 = performs fully), yielding a maximum possible total score of 66. Higher scores indicate better upper-limb motor function.

#### 2.6.2 Secondary outcomes

Activities of daily living were evaluated using the Modified Barthel Index (MBI) at the same time points. The MBI specifically assesses 10 essential ADL domains, including personal hygiene, bathing, feeding, toileting, stair climbing, dressing, bowel control, bladder control, chair/bed transfer, and ambulation. The total score ranges from 0 to 100, where higher scores denote a greater degree of functional independence (24).

To assess the success of participant blinding, subjects were asked at Week 3 to identify the type of intervention they believed they had received. The James Blinding Index (JBI) (25) was calculated to quantify blinding effectiveness, with values ranging from 0 (no blinding) to 1 (complete blinding), and 0.5 indicating random guessing.

All adverse events were recorded throughout the study. Each event was classified as treatment-related or unrelated based on its temporal and causal association with the intervention.

#### 2.6.3 EEG data acquisition

EEG recordings were obtained using a wireless 32-channel acquisition system (NeuroNexus, Shanghai Niantong Intelligent Technology Co., Ltd., China). The electrodes were positioned according to the international 10–20 system. Resting-state EEG data were collected under eyes-open conditions for 4 min at a sampling rate of 500 Hz. The ground electrode was placed at AFz, and the reference electrode was positioned at FCz. The electrode impedance was maintained below 20 kΩ.

#### 2.6.4 EEG data processing

##### 2.6.4.1 Preprocessing

EEG data were preprocessed using the EEGLAB toolbox (version 2021.0) implemented in MATLAB R2021a (The MathWorks Inc., Natick, MA, USA). The preprocessing steps included band-pass filtering (0.5–40 Hz), interpolation of bad channels, segmentation, rejection of noisy epochs, independent component analysis for artifact removal, and re-referencing to the common average.

##### 2.6.4.2 Resting-state functional network construction

**Figure 2:**
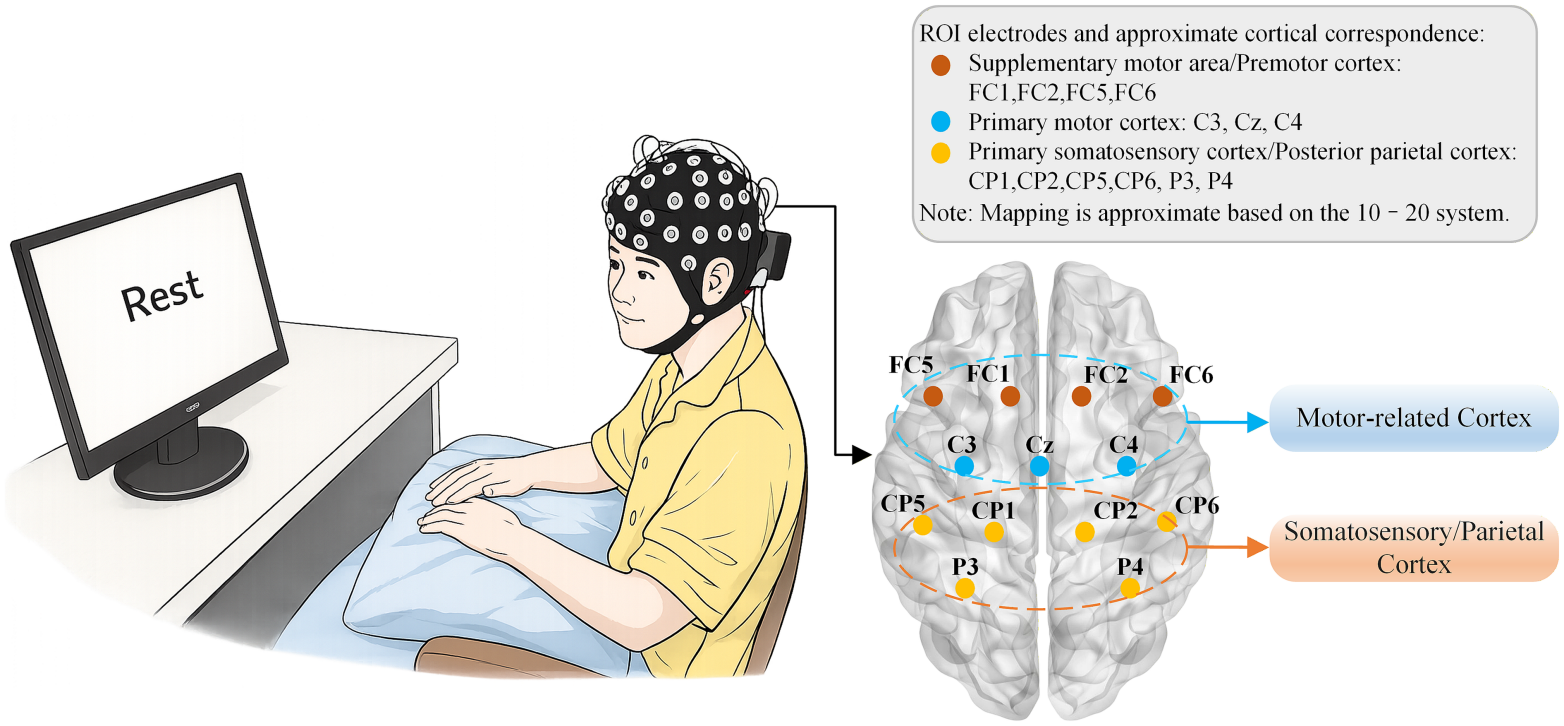
Schematic illustration of the EEG sensorimotor network regions of interest. Based on the international 10–20 system, FC1, FC2, FC5, and FC6 were defined as premotor/supplementary motor area-related electrodes; C3, C4, and Cz as primary motor electrodes; and CP1, CP2, CP5, CP6, P3, and P4 as primary somatosensory/posterior parietal electrodes. Cortical correspondence is approximate.


Based on previous findings (14), analyses focused on the alpha (8–13 Hz) and beta (13–30 Hz) frequency bands, where EA exhibits specific modulatory effects on the SMN. The region of interest (ROI) encompassed 13 electrodes corresponding to the SMN: FC1, FC5, C3, CP1, CP5, P3, Cz, FC2, FC6, C4, CP2, CP6, and P4. According to the international 10–20 system, these electrodes were grouped by their approximate functional cortical correspondence within the sensorimotor network (Figure 2). These electrodes were grouped as follows: FC1, FC2, FC5, and FC6 as premotor/supplementary motor area-related electrodes; C3, C4, and Cz as primary motor electrodes; and CP1, CP2, CP5, CP6, P3, and P4 as primary somatosensory/posterior parietal electrodes (26, 27). Whole-brain connectivity matrices were computed using the phase-locking value (PLV) algorithm, implemented via MATLAB-based custom scripts. To compute the PLV, the preprocessed EEG time series from each electrode were first band-pass filtered into the target alpha and beta bands. Next, the Hilbert transform was applied to extract the instantaneous phase, θ(t) , of each signal. The PLV between two signals *x* and *y* was then calculated to quantify their phase synchronization over *N* time points using the following formula:

$$\mathrm{PLV}=|\frac{1}{N}\sum_{t=1}^{N}e^{i(\phi_{x}(t)-\phi_{y}(t))}|$$

where $\phi_{x}(t)\;$ and $\phi_{y}(t)$ represent the instantaneous phases of signals *x* and *y* at time *t*. The resulting PLV is a normalized measure ranging from 0 (indicating completely random phase differences) to 1 (indicating perfect phase locking) (28).

##### 2.6.4.3 Graph theoretical analysis

In each PLV matrix, the electrodes represented network nodes and functional connections were defined as edges. Graph metrics were computed using the GRETNA toolbox in MATLAB. Prior to analysis, PLV connectivity matrices were binarized, and sparsity thresholds ranging from 5 to 50% (in 5% increments) were applied to retain the strongest connections between nodes. To normalize small-worldness and ensure robust estimation of network properties, 100 random networks preserving the same number of nodes, edges, and degree distribution as the original networks were generated for comparison.

**Figure 3:**
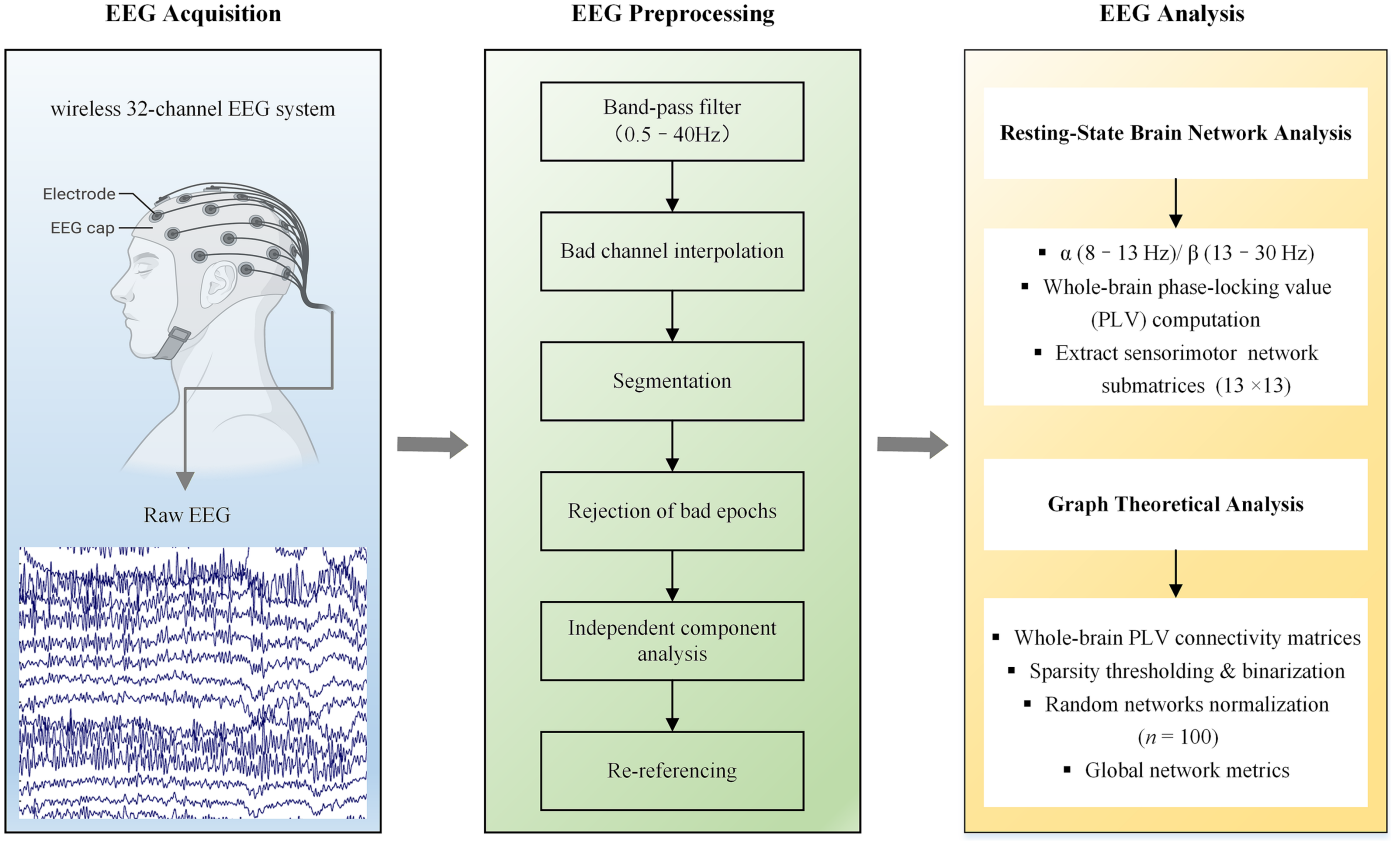
Flowchart of the EEG preprocessing and analysis pipeline. The workflow includes raw EEG acquisition, continuous EEG preprocessing, and resting-state network analysis. Functional connectivity was quantified using phase-locking value (PLV), followed by extraction of the 13 × 13 sensorimotor network submatrix and graph-theoretical analysis under sparsity thresholding. EEG, electroencephalography; PLV, phase-locking value.

Whole-brain PLV connectivity matrices were initially constructed in both frequency bands across all analyzed EEG electrodes. For functional connectivity analyses targeting the sensorimotor network, SMN submatrices (13 × 13) were subsequently extracted from the whole-brain matrices before and after treatment. In contrast, graph-theoretical analyses of global network topology were performed on the whole-brain PLV matrices, from which the clustering coefficient, characteristic path length, and small-worldness were calculated across a range of sparsity thresholds. The overall EEG preprocessing and analysis workflow is illustrated in Figure 3.

### 2.7 Statistical analysis

All statistical analyses were performed using SPSS version 26.0 (IBM Corp., Armonk, NY, USA). Behavioral data were analyzed based on the intention-to-treat (ITT) principle, including all randomized participants who completed at least 1 week of intervention. Missing behavioral data were imputed using the last observation carried forward (LOCF) method. In contrast, per-protocol (PP) analyses were applied for EEG and correlation analyses to ensure data completeness and reliability, as paired connectivity matrices are strictly required for within-group network comparisons.

The normality of all continuous variables was tested using the Shapiro–Wilk test. Data following a normal distribution were expressed as mean ± SD, whereas non-normally distributed data were presented as median (interquartile range). Categorical variables (e.g., sex, stroke type) were summarized as frequencies and compared using the chi-square (*χ^2^*) test.

Longitudinal behavioral outcomes were analyzed using linear mixed-effects models. This approach was chosen to account for repeated observations, baseline imbalance, and missing longitudinal data more robustly than multiple pairwise tests or traditional repeated-measures ANOVA. Baseline FMA-UE or MBI scores were included as covariates, and group, time, and the group × time interaction were entered as fixed effects. A random intercept was specified for each participant to account for within-subject correlation, and an autoregressive [AR (1)] covariance structure was used for repeated measures. Model parameters were estimated using restricted maximum likelihood. Furthermore, for between-group comparisons of clinical improvement (Δ scores), the independent-samples t-test was applied to normally distributed data, whereas the Mann–Whitney U test was used for non-normally distributed data. Where appropriate, Cliff’s delta (*δ*) was calculated as the effect-size measure (29).

Resting-state brain network analyses were conducted using the Network-Based Statistic (NBS) toolbox, which was also employed for multiple comparison corrections. For network-level analyses, a one-tailed test was applied within the NBS framework to detect direction-specific FC enhancement. For global graph-theoretical metrics (e.g., the area under the curve (AUC) of clustering coefficient, characteristic path length, and small-worldness), a two-way repeated-measures analysis of variance (ANOVA) was conducted to evaluate the main effects of Group and Time, as well as the Group × Time interaction.

The associations between changes in resting-state FC and upper-limb motor recovery were examined using Spearman’s rank correlation. For multivariate modeling, a data-driven pipeline was implemented to predict ΔFMA-UE using post-treatment FC. Predictor selection was performed via least absolute shrinkage and selection operator regression in RStudio (v4.3.2), utilizing leave-one-out cross-validation to determine the optimal penalty parameter ( λ _min). Retained features with non-zero coefficients were subsequently entered into a partial least squares regression (PLSR) model using SIMCA (v14.1; Sartorius Stedim Data Analytics AB, Sweden). A 7-fold cross-validation was applied during PLSR to identify the optimal number of latent components and validate predictive performance ( $Q^{2}$ ).

All other tests were two-tailed, and statistical significance was set at *p* < 0.05.

## 3 Results

### 3.1 Participants

**Figure 4:**
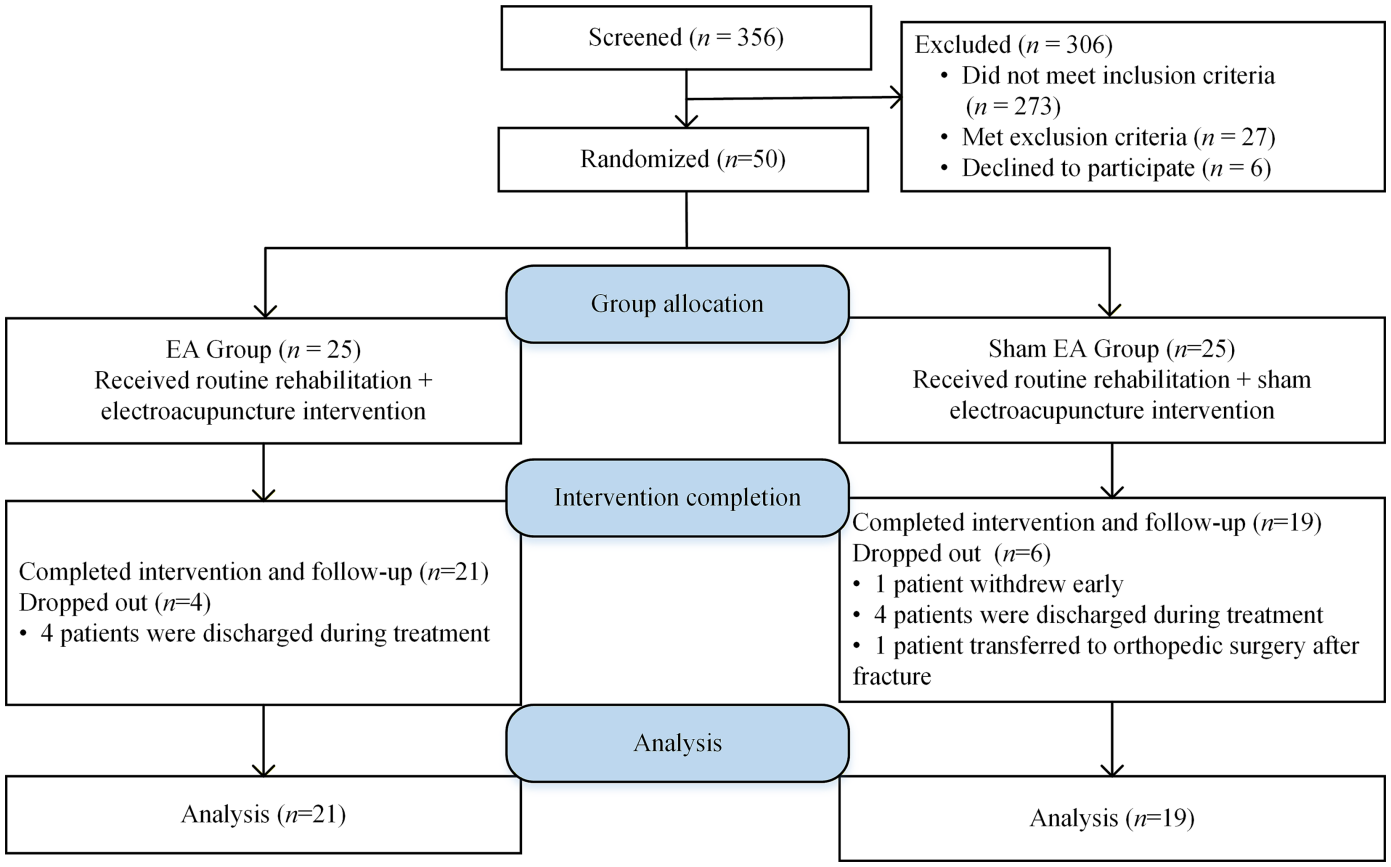
Participant flow diagram of the trial. A total of 356 patients were screened, 50 were randomized, and 40 completed the study and were included in the final analysis (EA group, *n* = 21; SEA group, *n* = 19). EA, electroacupuncture; SEA, sham electroacupuncture.

The recruitment flow is illustrated in Figure 4. Of the 356 screened patients, 50 met the inclusion criteria and were randomly assigned to the EA group (*n* = 25) or the SEA group (*n* = 25). After completing the intervention, 21 participants in the EA group and 19 in the SEA group underwent EEG data acquisition and were included in the final analysis. The dropout rates did not differ significantly between the groups (*p* > 0.05), indicating comparable retention (Table 1).

**Table 1:** Comparison of completion and dropout between groups (*n* = 50).

Outcomes	EA group	SEA group	*χ^2^*	*p*
Completed	21	19	0.5	0.48
Dropped out	4	6		

EA, electroacupuncture; SEA, sham electroacupuncture. Values are presented as number of participants.

The baseline demographic and clinical characteristics are summarized in Table 2. There were no significant differences between the two groups across any demographic or clinical metrics, specifically including sex, age, time post-stroke, stroke type, affected limb, and lesion location (*p* > 0.05).

**Table 2:** Demographic and clinical characteristics of participants (*n* = 50).

Variables	EA group (*n* = 25)	SEA group (*n* = 25)	*t*/*Z*/*χ^2^*	*p*
Sex (female/male)	14/11	13/12	0.081	0.777
Age (years)	52.24 ± 11.48	55.64 ± 9.06	−1.161	0.251
Time *p*ost-stroke (days)	22 (18)	21 (23)	−0.194	0.846
Stroke type (ischemic/hemorrhagic)	15/10	17/8	0.347	0.556
Affected limb (left/right)	10/15	14/11	1.282	0.258
Lesion location			0.000	1.000
Subcortical (basal ganglia, thalamus, corona radiata, internal capsule)	21	21		
Brainstem	2	2		
No MRI available	2	2		

EA, electroacupuncture; SEA, sham electroacupuncture; IQR, interquartile range. Values are presented as mean ± SD, median (IQR), or number of cases.

### 3.2 Clinical behavioral assessments

**Figure 5:**
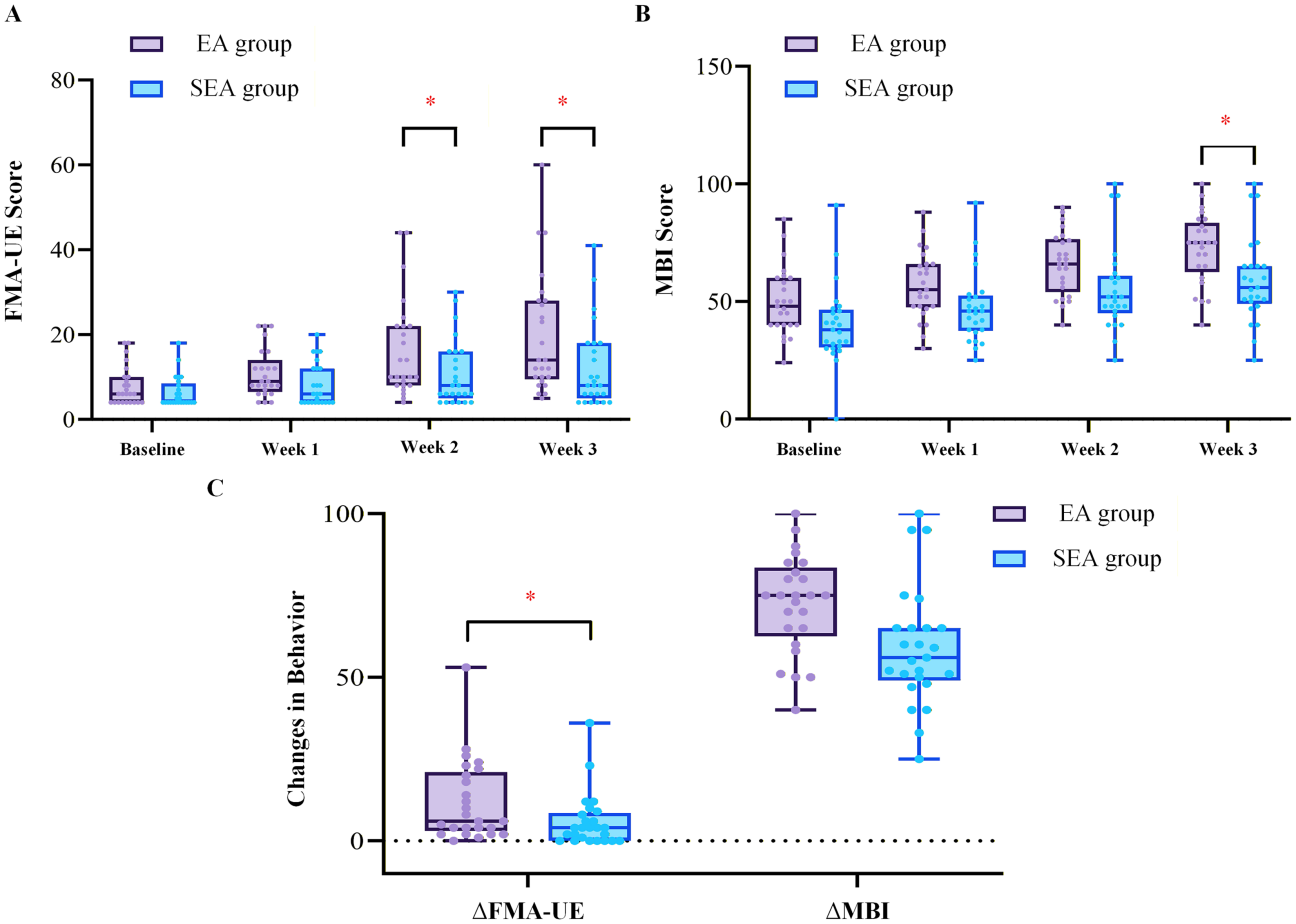
Longitudinal changes in behavioral outcomes. **(A,B)** FMA-UE and MBI scores at baseline and Weeks 1–3 in the EA and SEA groups. **(C)** Distribution of clinical improvements (ΔFMA-UE and ΔMBI, Week 3 minus baseline) in both groups. All data are presented as grouped boxplots with individual data points. In the boxplots, the center line indicates the median, boxes indicate the interquartile range (IQR), and the whiskers indicate the minimum and maximum values. Horizontal brackets indicate between-group comparisons (*p < 0.05). EA, electroacupuncture; SEA, sham electroacupuncture; FMA-UE, Fugl-Meyer Assessment for Upper Extremity; MBI, Modified Barthel Index.

For FMA-UE, the linear mixed-effects model revealed significant main effects of Time (*F* = 31.483, *p* < 0.001) and Group (*F* = 4.249, *p* = 0.045), alongside a significant Group × Time interaction (*F* = 4.243, *p* = 0.017). This significant interaction indicates distinct motor recovery trajectories between the two interventions. Post-hoc analyses demonstrated that the EA group achieved significantly higher FMA-UE scores than the SEA group at Weeks 2 and 3 (Figure 5A). From baseline to the endpoint, the median FMA-UE score increased from 6 to 14 in the EA group (*δ* = −0.654) and from 4 to 8 in the SEA group (*δ* = −0.414).

For MBI, significant main effects were observed for Time (*F* = 89.186, *p* < 0.001) and Group (*F* = 6.581, *p* = 0.013), although the Group × Time interaction was not significant (*F* = 1.947, *p* = 0.148). *Post-hoc* comparisons showed that MBI scores in the EA group were significantly higher than those in the SEA group at Week 3 (Figure 5B). Both groups exhibited substantial within-group improvements over the 3-week period (EA group *δ* = −0.715; SEA group *δ* = −0.643).

Furthermore, between-group analyses regarding the magnitude of clinical improvement (Δscores) revealed that the EA group achieved significantly greater gains in ΔFMA-UE compared to the SEA group (*Z* = −2.227, *p* = 0.026, Cliff’s *δ* = 0.365). In contrast, the difference in ΔMBI between the two groups was not statistically significant (*Z* = −1.224, *p* = 0.221, Cliff’s *δ* = 0.202) (Figure 5C).

### 3.3 Functional connectivity of the sensorimotor network

**Figure 6:**
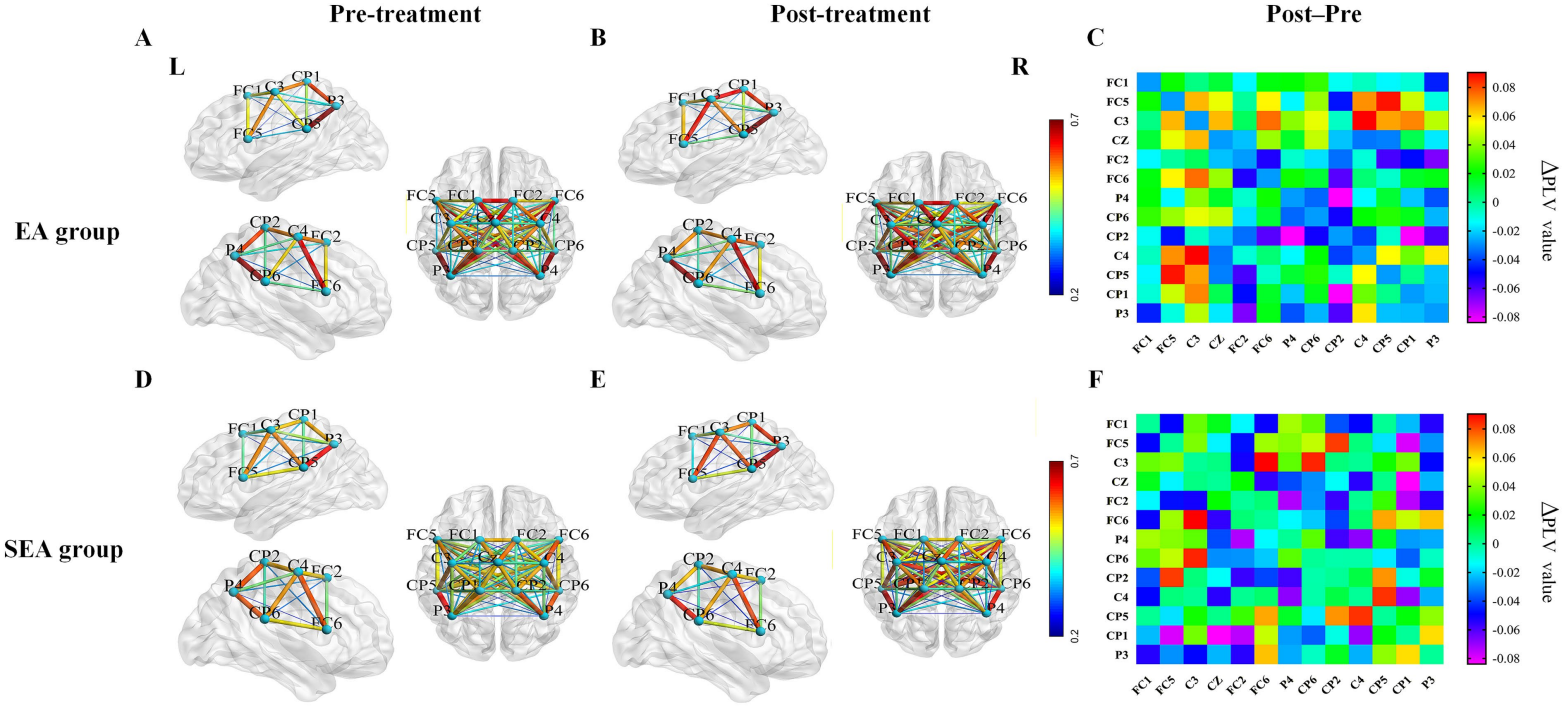
Alpha-band functional connectivity (FC) changes within the sensorimotor network (SMN) for the EA and SEA groups. **(A–C)** Averaged SMN connectivity maps of the EA group at pre-treatment and post-treatment, alongside the corresponding FC difference heatmap (post-treatment minus pre-treatment). **(D–F)** Corresponding connectivity maps and FC difference heatmaps for the SEA group. In the brain network maps, colored lines represent FC strength between electrode pairs, with warmer colors indicating stronger connections according to the adjacent color scale. In the heatmaps, each square represents the mean change in FC (ΔPLV) between two electrodes, where warmer colors denote greater positive FC differences. EA, electroacupuncture; FC, functional connectivity; L, left hemisphere; PLV, phase-locking value; R, right hemisphere; SEA, sham electroacupuncture; SMN, sensorimotor network.

**Figure 7:**
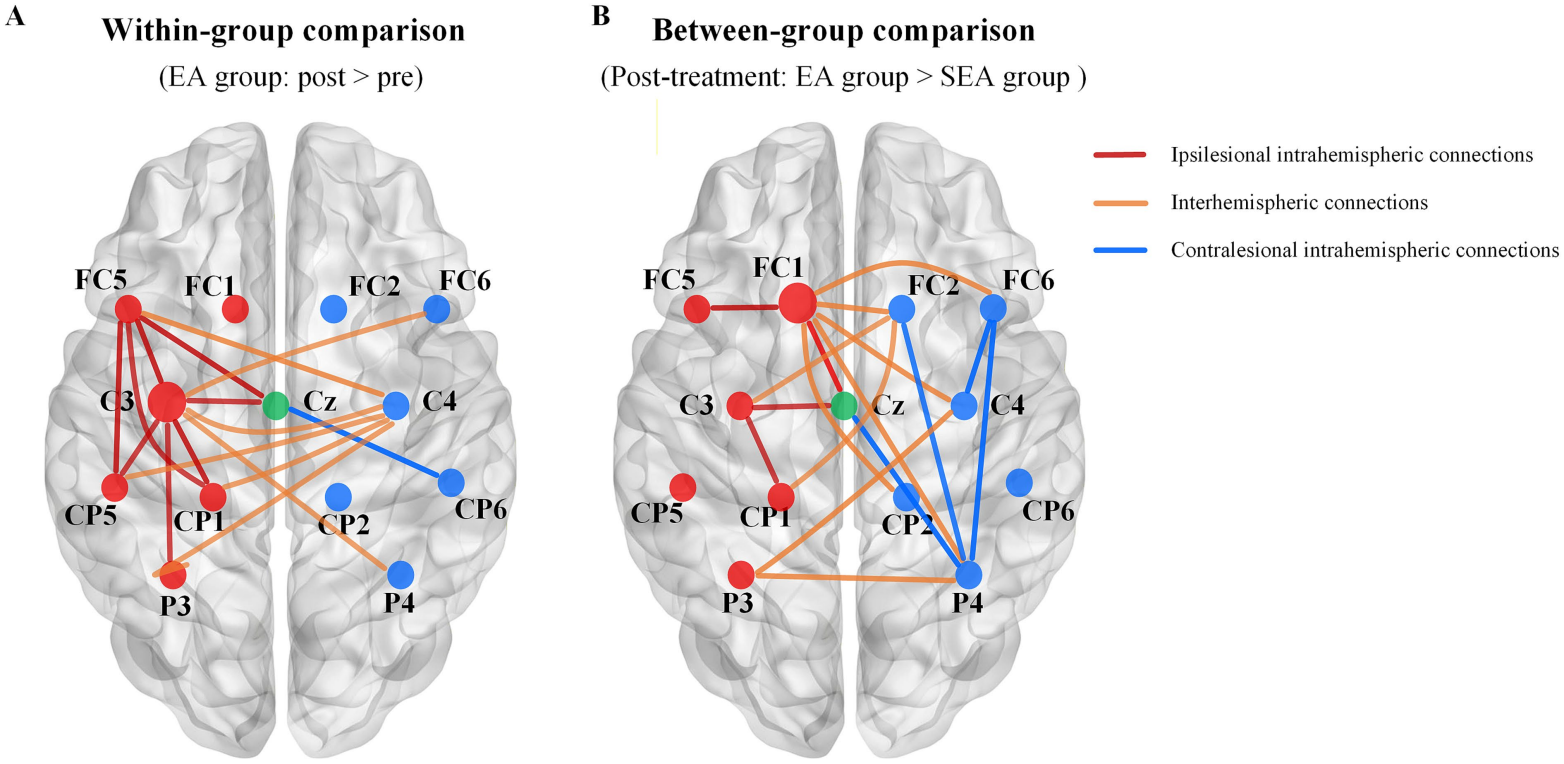
Significant alpha-band sensorimotor network (SMN) subnetworks identified by Network-Based Statistic (NBS) analysis. **(A)** Significant subnetwork for the within-group comparison in the EA group (post > pre). **(B)** Significant subnetwork for the between-group comparison at post-treatment (EA group > SEA group). Red nodes indicate ipsilesional electrodes, blue nodes indicate contralesional electrodes, and the green node represents the midline electrode (Cz). The enlarged nodes (C3 in panel **A**, FC1 in panel **B**) indicate the primary hubs with the highest number of significant connections in each subnetwork. The displayed networks are unweighted and binary. Connecting lines denote electrode pairs that showed significant connectivity differences after NBS correction. Red lines denote ipsilesional intrahemispheric connections, blue lines denote contralesional intrahemispheric connections, and orange lines denote interhemispheric connections. NBS, Network-Based Statistic; EA, electroacupuncture; SEA, sham electroacupuncture; SMN, sensorimotor network.

Brain connectivity maps and heatmaps revealed an overall increase in alpha-band FC within the SMN following EA treatment (Figures 6A–C), whereas no obvious changes occurred in the SEA group (Figures 6D–F). NBS analysis confirmed this significant within-group enhancement in the EA group (NBS-corrected *p* < 0.05). As illustrated in Figure 7A, the EA group exhibited enhanced FC across 16 electrode pairs. The subnetwork topology was predominantly driven by the enlarged ipsilesional central hub C3, comprising 8 intrahemispheric pairs (C3–FC5, C3–Cz, C3–CP5, C3–CP1, C3–P3, FC5–Cz, FC5–CP5, FC5–CP1) and 7 interhemispheric pairs (C4–FC5, C3–FC6, C3–P4, C3–C4, C4–CP5, C4–CP1, C4–P3).

Between-group analysis further demonstrated that post-treatment alpha-band SMN FC was significantly higher in the EA group than in the SEA group (NBS-corrected *p* < 0.05; Figure 7B). The enhanced subnetwork comprised 17 electrode pairs, distributed across bilateral and interhemispheric SMN regions. Notably, the topological visualization explicitly highlights the ipsilesional node FC1 as a prominent core hub driving these group differences. A dense cluster of nine pairs was concentrated in interhemispheric connections (FC1–FC2, FC1–FC6, FC1–P4, FC1–CP2, FC1–C4, C3–FC2, CP1–FC2, P3–P4, and C4–P3). No significant differences were detected in the beta band, either within or between groups (all *p* > 0.05).

### 3.4 Correlation between sensorimotor network connectivity and upper-limb motor recovery

**Figure 8:**
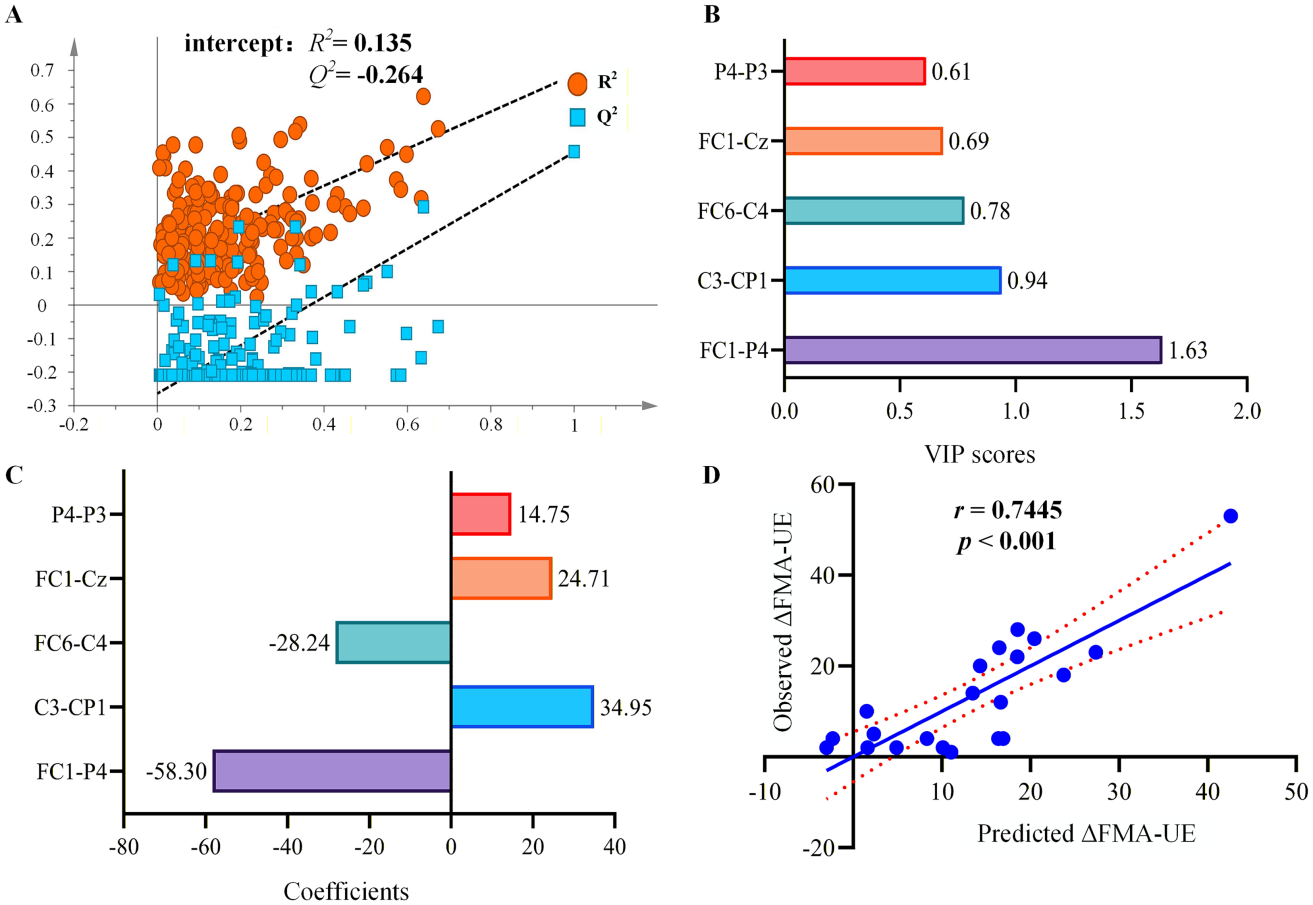
PLSR model performance and feature interpretation. **(A)** Permutation test results assessing the robustness and goodness of fit of the PLSR model. **(B)** Variable importance in projection (VIP) scores of the selected post-treatment FC predictors. **(C)** Regression coefficients of the selected FC predictors. Positive coefficients indicate positive associations with motor improvement, whereas negative coefficients indicate inverse associations. **(D)** Correlation between the predicted and observed ΔFMA-UE values in the EA group (*r* = 0.7445, *p* < 0.001). PLSR, partial least squares regression; EA, electroacupuncture; FC, functional connectivity; VIP, variable importance in projection; FMA-UE, Fugl-Meyer Assessment for Upper Extremity.

In the EA group, five predictors were selected from seventeen variables using LASSO regression, which were subsequently used to construct the PLSR model. The resulting model yielded a coefficient of determination ( $R^{2}$ ) of 0.687 and a root mean square error (RMSE) of 7.68. Permutation tests (Figure 8A) confirmed the robustness of the model, as the $R^{2}$ values from the permuted models were consistently lower than those of the original model. In contrast, no valid predictors were identified in the SEA group.

In the EA model, variable importance in projection (VIP) scores were used to quantify the contribution of each predictor to motor recovery. As shown in Figure 8B, the top five predictors ranked by the VIP value were FC1–P4, C3–CP1, FC6–C4, FC1–Cz, and P4–P3. Among these, FC1–P4 and FC6–C4 exhibited negative regression coefficients, whereas C3–CP1, FC1–Cz, and P4–P3 showed positive coefficients (Figure 8C). Notably, FC1–P4 displayed a significant negative correlation with FMA-UE improvement (*r* = −0.59, *p* = 0.005). Additionally, the predicted FMA-UE values derived from the EA model were significantly correlated with the observed values (*r* = 0.7445, *p* < 0.001; Figure 8D), demonstrating high predictive accuracy and clinical relevance.

### 3.5 Brain network topological properties

A two-way repeated-measures ANOVA showed no significant main effects of Time or Group, and no significant Group × Time interactions, for graph-theoretical metrics, including the AUC of clustering coefficient, characteristic path length, and small-worldness, in either the alpha or beta frequency bands (all *p* > 0.05). The detailed statistical results of these topological AUC metrics are comprehensively summarized in Supplementary Table S1.

### 3.6 Blinding assessment

The blinding assessment conducted at the end of the 3-week intervention period yielded a James Blinding Index (JBI) of 0.56 (95% CI: 0.41–0.70). According to established interpretive criteria, a JBI between 0.5 and 0.7 indicates effective participant blinding and randomization.

### 3.7 Adverse events

Only one adverse event was reported during the intervention period. One participant experienced a knee fracture caused by an accidental fall during hospitalization and was subsequently withdrawn from the trial for surgery. An independent safety evaluation determined that this event was unrelated to the study intervention. No treatment-related adverse effects, such as bleeding, infection, or persistent pain at the needle site, were observed in either group.

## 4 Discussion

In the current study, we investigated the clinical efficacy of EA for upper-limb motor recovery and its neuromodulatory effects on SMN reorganization in patients with subacute stroke. Our findings demonstrate that EA intervention, when added to conventional rehabilitation, significantly enhanced upper-limb motor function compared to sham EA. More importantly, this behavioral benefit was accompanied by selective enhancement of alpha-band FC within the SMN, particularly across ipsilesional intrahemispheric and interhemispheric sensorimotor regions. Regression analyses further indicated that post-treatment connectivity patterns were associated with the magnitude of motor improvement. Taken together, these findings suggest that EA may facilitate post-stroke recovery not only through nonspecific sensory stimulation, but through frequency-specific reorganization of recovery-relevant sensorimotor circuits.

### 4.1 EA improves upper-limb motor recovery in subacute stroke

Across 3 weeks of intervention, the EA group exhibited significantly greater FMA-UE gains than the SEA group, with a meaningful effect size, supporting the clinical efficacy of EA as an adjunct to standard rehabilitation. This result is consistent with the growing body of evidence supporting EA-mediated promotion of post-stroke motor recovery (30). The absence of severe adverse events and comparable dropout rates between groups further supports the safety and feasibility of the protocol in this patient population.

In contrast, post-treatment MBI scores did not differ significantly between groups, despite both groups showing marked within-group improvements. This discrepancy may reflect the different constructs captured by the two scales. FMA-UE is a relatively specific measure of upper-limb motor impairment, whereas MBI reflects broader independence in activities of daily living and is influenced by multiple factors, including trunk control, lower-limb function, balance, cognition, and environmental support (31). Therefore, improvements in upper-limb motor impairment over a 3-week intervention may not immediately translate into proportional gains in global daily living ability. These findings suggest that EA may exert a more direct short-term effect on upper-limb motor recovery, whereas its impact on functional independence may require longer observation or more comprehensive rehabilitation targets.

### 4.2 EA selectively reorganizes alpha-band sensorimotor connectivity in subacute stroke

The central neurophysiological finding of this study was the selective enhancement of alpha-band SMN connectivity after EA. This is relevant because post-stroke recovery is increasingly understood as a process of circuit-level reorganization rather than focal lesion repair alone. Stroke disrupts communication within and between sensorimotor regions, and recovery depends in part on how surviving circuits are reorganized over time rather than simply preserved structurally (32–34). Within this framework, the present pattern suggests that EA promoted a more coordinated sensorimotor network state, especially across ipsilesional and interhemispheric nodes relevant to motor recovery.

From a mechanistic perspective, alpha oscillations do not merely reflect cortical “idling” but function as an active “gating by inhibition” mechanism (35). Experimental and theoretical work suggests that alpha activity contributes to sensory gating, suppression of task-irrelevant activity, and stabilization of interregional communication (36, 37). In sensorimotor systems, prestimulus alpha dynamics are closely related to cortical excitability (38). Recent studies suggest that alpha oscillations shape perceptual sensitivity and influence the neural representation of incoming somatosensory stimuli (39). Accordingly, the enhanced alpha-band coupling observed here may reflect a more efficient sensorimotor processing state in which relevant communication is reinforced while competing or inefficient activity is better constrained.

The present findings are also consistent with prior EEG and magnetoencephalography (MEG) studies of stroke recovery. Resting-state studies increasingly suggest that alpha-band sensorimotor connectivity is related to upper-limb status and recovery trajectories after stroke. Using directed functional connectivity analysis, Pirovano et al. reported that successful motor rehabilitation was associated with strengthened ipsilesional intrahemispheric connectivity together with reduced contralesional motor connectivity (13). Similarly, Tang et al. further showed that greater recruitment of an ipsilesional alpha-band resting-state sensorimotor network during the subacute stage was associated with better concurrent motor performance (40). Together, these observations support the view that alpha-band SMN reorganization is behaviorally meaningful and plausibly adaptive in the recovering post-stroke brain.

A notable observation was that both groups showed behavioral improvement, whereas significant alpha-band SMN reorganization was observed only in the EA group. This dissociation suggests that behavioral recovery and electrophysiological network reorganization may not fully overlap. Because both groups received conventional rehabilitation during the subacute phase, some spontaneous and training-related improvement would be expected (33). However, active EA may provide an additional and more structured afferent drive to the sensorimotor system compared with sham EA (12). In the present protocol, EA delivered repeated electrical stimulation through selected upper-limb acupoints with defined frequency, intensity, duration, and polarity, whereas sham EA involved superficial needling without effective current stimulation. Such patterned peripheral input may more effectively engage somatosensory afferent pathways and repeatedly activate sensorimotor cortical circuits (41), thereby facilitating alpha-band SMN reorganization beyond nonspecific rehabilitation-related gains. Thus, the selective increase in alpha-band connectivity after EA may reflect a treatment-specific form of network adaptation rather than a generic correlate of behavioral improvement alone.

### 4.3 EA-related motor recovery depends on the pattern of connectivity reorganization

The PLSR analysis suggests that the clinical relevance of SMN reorganiz lies in the specific pattern of connectivity reorganization rather than in a global increase in connectivity alone. In the EA group, strengthened functionally specific connections, particularly C3–CP1, FC1–Cz, and P4–P3, were positively associated with motor gains. Among these connections, C3–CP1 and FC1–Cz involve or approximate ipsilesional motor, somatosensory, premotor, and midline motor-related regions that are central to sensorimotor integration and fine motor control. Their strengthening may therefore reflect a more focused and functionally relevant reorganization of perilesional communication. Furthermore, the positive contribution of P4–P3 may indicate more coordinated bilateral posterior sensorimotor or parietal integration, which may be relevant for proprioceptive processing and spatial representation of the affected limb. This interpretation is consistent with prior transcranial magnetic stimulation (TMS) and transcranial direct current stimulation (tDCS) studies showing that better motor recovery is generally associated with stronger ipsilesional connectivity and more balanced interhemispheric organization (42, 43).

By contrast, enhanced long-range connections such as FC1–P4 and FC6–C4 were negatively associated with motor improvement. These connections span interhemispheric or contralesional sensorimotor regions and may reflect a more diffuse compensatory configuration. Current stroke literature suggests that contralesional recruitment is not uniformly maladaptive; rather, its significance depends on impairment severity, structural reserve, and recovery stage (44, 45). In mildly affected patients with preserved ipsilesional structural resources, restoring interhemispheric balance may be favorable (46), whereas in more severely affected patients contralesional support may temporarily contribute to compensation (47, 48). The present negative associations therefore suggest that, in our cohort, greater reliance on these long-range interactions was linked to less favorable recovery, whereas more focused ipsilesional and balanced interhemispheric coupling appeared more adaptive. This pattern suggests that EA may share recovery-relevant network targets with established central neuromodulation approaches, such as enhancing ipsilesional sensorimotor integration and restoring interhemispheric balance. However, unlike TMS or tDCS, which directly modulate cortical excitability through electromagnetic or transcranial electrical stimulation, EA may influence these networks through a bottom-up mechanism driven by repeated somatosensory afferent input.

### 4.4 Translational implications

From a translational perspective, the present findings support the potential value of EEG-based alpha-band SMN connectivity as a candidate biomarker for monitoring EA efficacy. Because this measure was selectively modulated by active EA and was associated with motor improvement, it may provide a physiologically meaningful index of treatment responsiveness. More broadly, longitudinal EEG measures may complement conventional behavioral scales by capturing recovery-related neural reorganization that is not directly visible at the clinical level.

At the same time, the clinical translation of such measures should be viewed cautiously. Although the present regression analyses indicate that post-treatment connectivity patterns were informative for recovery magnitude, they do not establish whether these patterns directly mediate behavioral improvement. Accordingly, alpha-band SMN connectivity should be regarded at present as a candidate monitoring marker rather than a validated decision-making biomarker. Further longitudinal and mechanistic studies are needed to determine whether this measure can reliably track treatment response across different patient profiles and recovery stages.

Nevertheless, these findings suggest several practical directions for precision neurorehabilitation. Repeated EEG assessment may help identify patients who are more likely to show recovery-relevant network reorganization after EA and provide a feasible means of tracking intervention-related plasticity during the subacute phase. If validated prospectively, such neurophysiological signatures may also support more individualized treatment strategies, including adjustment of EA parameters according to each patient’s ongoing sensorimotor oscillatory state. This possibility is conceptually consistent with emerging brain-state-dependent and closed-loop neuromodulation approaches.

### 4.5 Methodological considerations and limitations

Several methodological considerations should be acknowledged. First, although this study adopted a randomized controlled design, the relatively small sample size may have limited the statistical power to detect subtle between-group differences, particularly in secondary outcomes. Future studies with larger, multicenter cohorts are warranted to validate the present findings and improve generalizability. Second, our trial reports only immediate post-treatment outcomes at the conclusion of the 3-week intervention. The absence of long-term follow-up limits our ability to evaluate the durability of these treatment effects on motor recovery and cortical reorganization. Given that neuroplastic adaptations may continue to evolve beyond the 3-week intervention period, future longitudinal studies incorporating extended clinical and neuroimaging assessments are essential to capture the sustainability of EA-induced neural modulation. Third, while EEG provides superior temporal resolution for characterizing neural synchronization, its spatial specificity remains limited compared with imaging modalities such as functional magnetic resonance imaging or magnetoencephalography. Multimodal neuroimaging approaches may therefore provide a more comprehensive understanding of the spatial and temporal properties of EA-induced brain network reorganization. Finally, individual variability in stroke lesion location, lesion burden, and recovery stage may have influenced both treatment responsiveness and network reorganization. Future studies should incorporate stratified analyses based on lesion characteristics and employ personalized stimulation protocols to optimize therapeutic outcomes.

## 5 Conclusion

In conclusion, EA combined with conventional rehabilitation elicited significantly greater improvements in upper-limb motor recovery than sham EA in subacute stroke. These behavioral gains were accompanied by selective enhancement of alpha-band FC within the SMN, particularly involving ipsilesional intrahemispheric and interhemispheric sensorimotor pathways. Furthermore, specific post-treatment connectivity patterns were associated with the magnitude of motor improvement, suggesting that EA-related motor recovery may involve frequency-specific and circuit-level reorganization of sensorimotor networks. Together, these findings provide neurophysiological evidence for EA as a safe and potentially effective adjunctive intervention in post-stroke motor rehabilitation. Future longitudinal, large-scale, and multimodal studies are warranted to further clarify the temporal dynamics and mechanistic pathways of EA-induced neuroplasticity.

## Data Availability

The raw data supporting the conclusions of this article will be made available by the authors, without undue reservation.
