# Supplementary material for: Electroacupuncture improves upper-limb motor function and modulates sensorimotor network connectivity in subacute stroke: a randomized controlled trial
Source: Front Neurol. 2026 Jun 19;17:1780918. doi: 10.3389/fneur.2026.1780918 (PMC13328037; doi:10.3389/fneur.2026.1780918)
Supplement: Supplementary file 1 [file Table_1.docx]

Supplementary Material

Electroacupuncture Improves Upper-Limb Motor Function and Modulates Sensorimotor Network Connectivity in Subacute Stroke: A Randomized Controlled Trial

Mingfen Li^a^, Weigeng Zou^a^, Genggeng Xie^a^, Man Li^a^, Su Zheng^a^, Haifeng Li^a,*^

*** Correspondence:** 371955754@qq.com

# Supplementary Material

Table S1. Comparison of global network topology metrics in the alpha and beta bands (*n* = 40)

| **Band** | **Metric** | **Time** | | **Group** | | **Time × Group** | |
| --- | --- | --- | --- | --- | --- | --- | --- |
|  |  | *F* | *p* | *F* | *p* | *F* | *p* |
| Alpha | Clustering coefficient | 0.049 | 0.827 | 0.140 | 0.710 | 0.779 | 0.383 |
|  | Characteristic path length | 0.241 | 0.626 | 0.115 | 0.736 | 0.020 | 0.889 |
|  | Small-worldness | 0.251 | 0.619 | 2.180 | 0.148 | 0.147 | 0.704 |
| Beta | Clustering coefficient | 0.016 | 0.900 | 1.908 | 0.175 | 0.034 | 0.854 |
|  | Characteristic path length | 1.149 | 0.290 | 2.594 | 0.116 | 1.128 | 0.295 |
|  | Small-worldness | 0.432 | 0.515 | 0.131 | 0.719 | 3.069 | 0.088 |

Note: *F* and *p* values were derived from the two-way repeated-measures ANOVA. *n* = 40 represents the per-protocol analysis set for EEG data (21 in the EA group and 19 in the SEA group).
